# Changes in separate renal function in patients who underwent minimally invasive renal stone surgery according to the preoperative functional deterioration

**DOI:** 10.1038/s41598-019-40485-x

**Published:** 2019-03-05

**Authors:** Min Soo Choo, Juhyun Park, Min Chul Cho, Hwancheol Son, Hyeon Jeong, Sung Yong Cho

**Affiliations:** 1 Department of Urology, Hallym University Dongtan Sacred Heart Hospital, Hwaseong, Korea; 2 Department of Urology, SMG-SNU Boramae Medical Center, Seoul, Korea; 3 Seoul National University Hospital, 101, Daehak-ro Jongno-gu, Seoul, 03080 South Korea

**Keywords:** Kidney, Urology

## Abstract

The significant predictors for the postoperative deterioration of separate renal function after minimally invasive stone surgery were investigated in the present prospective and observational study. A total of 117 consecutive patients who underwent retrograde intrarenal surgery or mini-percutaneous nephrolithotomy for renal calculi >10 mm were included in the present study. Perioperative changes in separate renal function were evaluated with Technetium-99m-Diethylene TriaminePenta acetic acid scan prior to intervention and at postoperative 3 months. Based on the functional differences between bilateral renal units, deterioration of separate renal function was graded into the following three groups: normal deterioration (<10%), moderate deterioration (10–20%), and severe deterioration (>20%). A total of 46 patients had a normal separate renal function, while 71 (60.7%) showed abnormal separate function in the involved side, including 29 (24.8%) moderate and 42 (35.9%) severe deterioration. Postoperatively, 48 patients (41.0%) showed aggravation or no recovery of separate renal function. Of the 46 patients with normal separate function, only 9 patients (19.5%) showed postoperative aggravation. Patients with moderate and severe deterioration showed aggravation (n = 7, 24.1%) or no recovery of separate renal function (n = 32, 76.1%, *P* < 0.001). Preoperative severe deterioration of separate renal function was an independent significant predictor for the postoperative deterioration of renal function (OR: 9.09, 95% CI: 4.007–20.624, *P* < 0.001). Lower preoperative deterioration of separate renal function showed a high probability of functional recovery. Therefore, it is hypothesized that early intervention might be necessary in cases where the patient exhibits severe aggravation of renal function.

## Introduction

European Association of Urology guidelines have recommended the following indications for active removal of renal stones: growing stone, stones in high-risk stone formers, urinary tract obstruction, infection, stone-related symptoms, large-sized stones >15 mm, small stones if observation is not the option of choice, patients’ preference, underlying comorbidity, and patients’ social situation and persistent stones >2 to 3 years^1^. However, the criteria failed to include functional assessment, although the presence of urinary stones, one or more stone-related episodes, and underlying comorbidity are well-known risk factors of renal functional deterioration^2^.

Renal calculi can act as an aggravating factor of renal function^3,4^. Renal function is generally expected to recover after the removal of renal calculi^5,6^. However, in our previous study, it was observed that some patients with successful minimally invasive renal stone surgery did not exhibit any improvement from deterioration of renal function^7^. In previous studies, it has been suggested that preoperative deterioration of renal function is a risk factor associated with worsening or not recovering after surgery^8^. However, the reported studies used serum markers as indicators of renal function, which might not adequately reflect the function of the involved renal unit.

Recent studies have demonstrated the feasibility of mini-percutaneous nephrolithotomy (mini-PCNL) and retrograde intra-renal surgery (RIRS) as minimally invasive stone surgery^9^. Previously, we have reported our initial experience on changes in perioperative renal function by comparing relative renal function with deteriorated separate renal function in patients using diethylenetriamine pentaacetic acid (^99m^Tc-DTPA)^7^. Renal functional data are useful for discovering significant predictors of preoperative renal functional deterioration in patients with renal stones >10 mm. However, significant predictors with a potential to elucidate perioperative changes in renal functional deterioration, regardless of preoperative renal functional status have not been reported. Therefore, the objective of this study was to compare changes in perioperative separate renal function in patients in whom the main renal stone >10 mm was removed by mini-PCNL or RIRS, based on the evaluation by ^99m^Tc-DTPA scintigraphy. Also, the objective was to determine significant predictors with a potential to explain the postoperative deterioration of separate renal function in both a prospective and an observational study.

## Materials and Methods

### Patients and study design

After approval of the study design by the Institutional Review Board at the Seoul Metropolitan Government – Seoul National University Boramae Medical Center (16-2015-31) and using patient information stored in the hospital database, patients aged ≥20 years were included in this study. All patients provided written informed consent, and this study was performed in strict accordance with the ethical guidelines of the Declaration of Helsinki. Medical charts of consecutive patients who underwent RIRS or mini-PCNL were included in the prospective database. Active removal of renal stones was carried out by following the European Association of Urology guidelines^1^. Renal stones with a maximal diameter >10 mm, growing stones, stones in high-risk stone formers, and extracorporeal shock-wave lithotripsy-resistant stones were analyzed. Cases with bilateral stones, active infection, anatomical abnormality of musculoskeletal deformities, pre-stenting DJ catheter or ureteral strictures were excluded. Cases with hydronephrosis were included in the analysis unless there was definite evidence of complete obstruction.

### Surgical methods

Mini-PCNL procedures were similar to the procedures mentioned in the previous investigations^1^. Percutaneous nephrostomy was performed one day before or on the day of surgery. The flank area was upholstered by a long gel form to 30 degrees from the table. The percutaneous tract was dilated by an 18Fr Ultraxx Nephrostomy Balloon (Cook Medical, Bloomington, IN, USA). A 15-Fr Miniaturized Nephroscope (Richard Wolf, Knittlingen, Germany) was inserted into the renal pelvis. A double-J stent was routinely inserted before stone fragmentation to obstruct the ureter. An 80 W holmium laser (Trimedyne Inc., Irvine, USA) was used as a lithotrite. Stone forceps were not employed for extracting the fragmented stones because small fragments or dust waded out of the body spontaneously through the sheath with an endoscopy irrigation pump (Stryker, Michigan, USA). After the percutaneous procedure, flexible ureterorenoscope was inserted into the renal pelvis in the antegrade or retrograde manner to remove the remnant stones simultaneously. Nephrostomy tubes were not routinely used for tubeless procedures. The RIRS procedures were performed as described previously^10^.

### Clinical parameters

Patients’ age at the time of surgery, gender, body mass index, serum creatinine level, estimated glomerular filtration rates, and hemoglobin level were analyzed. Computed tomography (CT) scans were performed preoperatively for all patients. Stone-related parameters including treatment history, operative methods, surgical laterality, stone composition, stone hardness (Hounsfield units), maximal diameters, and stone volume were measured by CT scans. Stone volume was calculated by the sum of each stone (0.523 × length × width × height). Stone distribution was analyzed using the Seoul National University Renal Stone Complexity (S-ReSC) score which classified the collecting system into nine spaces of the renal pelvis, superior/inferior major calyces, and anterior/posterior minor calyces of the superior/middle/inferior calyces^11^. Follow-up low-dose CT images were acquired at postoperative 60–90 days. Stone-free status was clinically defined as absence of evidence of remnant stone >2 mm.

### Assessment of predictors for improvement of renal function

The preoperative renal function was assessed by eGFR and ^99m^Tc-DTPA nuclear medicine tests for all patients prior to surgery and at postoperative 60–90 days. According to the gap of separate renal function between the two kidneys, renal functional status was graded into 3 groups: <10% (normal), 10% to 20% (moderate deterioration), and >20% (severe deterioration). The measurement of the absolute individual kidney function using DTPA may be influenced by the patient’s current condition, especially the hydration status. And in the kidney with obstruction, the value is underestimated and can be inaccurate. In addition, measurements of the absolute individual kidney function have been suggested to be more inaccurate in patients with reduced total renal function. However, the comparison of the relative renal function is relatively well comparable regardless of the patient’s total kidney function or hydration status.

### Statistical analysis

Independent t-test or Mann-Whitney U test was performed to compare the results between the two groups. Chi-square and Fisher’s exact test were used to analyze categorical variables. Univariate and multivariate logistic regression analyses with backward stepwise selection were used to find significant predictors for improvement or aggravation of the separate renal function. Statistical significance was considered when the *P* value was less than 0.05. Statistical analyses were performed using IBM SPSS Statistics version 20 (IBM, Chicago, IL, USA) and R version 3.0.1 (http://www.r-project.org).

## Results

The characteristics of the patients and stones are summarized in Table 1. The mean age of the 117 patients was 57.3 ± 13.5 years. Stone-free rate was observed in 85.5% (100 of 117) of study population. The mean size of the remnant stones >2 mm was 5.7 ± 5.3 mm, which was managed by extracorporeal shock-wave lithotripsy in seven cases and ureteroscopic surgery in two cases. Complications occurred in 10 cases (8.5%). Two of the 10 cases showed postoperative ureteral strictures after 6 months, while three cases showed the incidence of postoperative fever and five cases revealed the onset of pain.

**Table 1:** Baseline characteristics of the patients and stones according to the preoperative functional status of separate renal function in the involved side.

	Total	Normal	Moderate	Severe	*P*
	117	46 (39.3)	29 (24.8)	42 (35.9)	
**Patient characteristics**					
Age (years)	57.3 ± 13.5	54.2 ± 13.9	59.4 ± 13.7	59.2 ± 12.4	0.140
Gender					0.581
Male	80 (68.4)	34 (73.9)	19 (65.5)	27 (64.3)	
Female	37 (31.6)	12 (26.1)	10 (34.5)	15 (35.7)	
Body mass index (kg/m^2^)	24.9 ± 3.6	24.1 ± 2.9	25.4 ± 3.3	25.3 ± 4.3	0.219
Creatinine (mg/dl)	0.99 ± 0.31	0.95 ± 0.33	1.02 ± 0.30	1.02 ± 0.26	0.430
Estimated GFR (mL/min/1.73 m^2^)	77.2 ± 22.4	85.3 ± 24.6	72.2 ± 20.7	71.6 ± 18.2	0.005
Diabetes	35 (29.9)	12 (26.1)	12 (41.4)	11 (26.2)	0.299
Hypertension	52 (44.4)	14 (30.4)	14 (48.3)	24 (57.1)	0.037
Prior ESWL	36 (30.8)	14 (30.4)	5 (17.2)	17 (40.5)	0.114
**Stone characteristics**					
Laterality					0.319
Right	46 (39.3)	19 (41.3)	14 (48.3)	13 (31.0)	
Left	71 (60.7)	27 (58.7)	15 (51.7)	29 (69.0)	
Number of stones	3.1 ± 3.2	3.3 ± 3.3	2.1 ± 1.9	3.5 ± 3.6	0.183
Maximal stone size (mm)	17.2 ± 13.6	14.8 ± 9.6	19.6 ± 16.2	18.2 ± 15.0	0.032
Total stone volume (mm^3^)	3345 ± 7115	2406 ± 5838	3987 ± 7228	3930 ± 8277	0.185
Hounsfield unit	879 ± 380	884 ± 356	983 ± 382	863 ± 413	0.945
Staghorn stone	11 (9.4)	3 (6.5)	2 (6.9)	6 (14.3)	0.399
Hydronephrosis without obstruction	43 (36.8)	9 (19.6)	11 (37.9)	23 (56.1)	0.002
S-ReSC scores for PCNL	2.4 ± 1.7	2.6 ± 1.7	2.3 ± 2.0	2.4 ± 1.6	0.759
S-ReSC scores for RIRS	3.6 ± 2.5	3.8 ± 2.3	3.4 ± 3.0	3.6 ± 2.5	0.718

Values are presented as mean ± standard deviation or number (%). GFR = glomerular filtration rate; ESWL = extracorporeal shockwave lithotripsy; S-ReSC = Seoul National University Renal Stone Complexity; PCNL = percutaneous nephrolithotomy; RIRS = retrograde intrarenal surgery.

**Figure 1:**
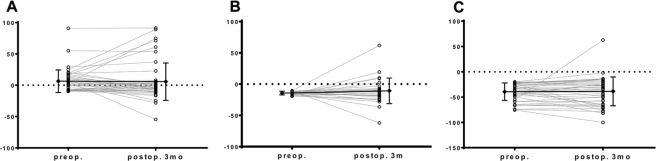
The student paired t-test of perioperative separate functional changes in the involved kidneys according to the preoperative functional status. a. normal (<10%), b. moderate (10–20%), and c. severe (>20%). Data are presented as individual values and as a mean ± standard deviation.

Perioperative separate renal function changes are shown in Table 2. About two-thirds of patients (71/117, 60.7%) showed abnormal separate renal function in the involved side, including 29 patients (24.8%) with moderate deterioration and 42 patients (35.9%) with severe deterioration. Of the 46 patients who showed normal separate function, only 9 patients (19.5%) showed postoperative aggravation. Of the 71 patients with abnormal separate renal function, aggravation was present in 18 (25.4%) moderate deterioration cases and 32 (45.1%, *P* < 0.001) severe deterioration cases. Changes from preoperative normal, moderate, and severe deterioration to postoperative severe deterioration were in 6.5%, 24.1% and 76.2% of cases, respectively (Fig. 1).

**Table 2:** Surgical outcomes according to the preoperative functional status of separate renal function in the involved side.

	Total	Normal	Moderate	Severe	*P*
	117	46 (39.3)	29 (24.8)	42 (35.9)	
Operative method					0.263
RIRS	97 (82.9)	38 (82.6)	23 (79.3)	36 (85.7)	
Mini-PCNL	20 (17.1)	8 (17.4)	6 (20.7)	4 (9.5)	
Main composition of the stone					0.376
Calcium oxalate monohydrate	64 (54.7)	26 (56.5)	14 (48.3)	24 (57.1)	
Calcium oxalate dehydrate	20 (17.1)	7 (15.2)	5 (17.2)	8 (19.0)	
Uric acid	25 (21.4)	11 (23.9)	7 (24.1)	7 (16.7)	
Carbonate apatite	8 (6.8)	2 (4.3)	3 (10.3)	3 (7.1)	
Operative parameters					
Mean operative time (min)	72.1 ± 57.0	66.7 ± 59.9	80.8 ± 61.6	71.8 ± 50.6	0.583
Mean estimated blood loss (ml)	14.7 ± 61.9	22.1 ± 90.8	17.2 ± 42.8	4.7 ± 18.5	0.409
Mean hospital day	1.7 ± 2.5	2.0 ± 2.9	1.0 ± 0.3	1.9 ± 2.6	0.234
Complications	10 (8.5)	6 (13.0)	1 (3.4)	3 (7.1)	0.207
Grade I	3 (2.6)	2 (4.3)		1 (2.4)	
Grade II	5 (4.3)	3 (6.5)	1 (3.4)	1 (2.4)	
Bleeding without embolization	4 (3.4)	2 (4.3)	1 (3.4)	1 (2.4)	
Fever more than 38.0 °C with antibiotics	1 (0.9)	1 (2.2)			
Grade III-V (stricture)	2 (1.7)	1 (2.2)		1 (2.4)	
Stone-free status	100 (85.5)	39 (84.8)	23 (79.3)	38 (90.5)	0.464

Values are presented as mean ± standard deviation or number (%). RIRS = retrograde intrarenal surgery; Mini-PCNL = miniaturized percutaneous nephrolithotomy.

The percentage of patients with preoperative normal separate renal function was 80.4% (37 of 46 patients) as shown in Table 3. Improvement from preoperative moderate and severe deterioration to postoperative normal separate renal function occurred in 37.9% (11 of 29) and 4.8% (2 of 42) cases, respectively (OR: 0.082, 95% CI: 0.016–0.408, *P* = 0.001). Regardless of the type of surgery, improvement rates remained the same.

**Table 3:** Perioperative changes in differences in separate renal function.

Differences in separate renal function		Status	Postoperative			Sum
			Normal (<10%)	Moderate (10–20%)	Severe (>20%)	
Preoperative	Normal (<10%)	n	37	6	3	46
		Maintained	80.4%			100%
		Aggravated		13.0%	6.5%	
	Moderate (10–20%)	n	11	11	7	29
		Improved	37.9%			100%
		Maintained		37.9%		
		Aggravated			24.1%	
	Severe (>20%)	n	2	8	32	42
		Improved	4.8%	19.0%		100%
		Not improved			76.2%	
Sum			50 (42.7%)	25 (21.4%)	42 (35.9%)	117 (100%)

Predictors for postoperative severe renal function deterioration were analyzed by univariate and multiple regression analyses and included (1) patient’s age, gender, body mass index, creatinine level, estimated GFR, diabetes, and hypertension, (2) stone factors of laterality, number of stones, maximal stone size, and hydronephrosis, and (3) operative characteristics of mean operative time, mean hospital day, stone-free status, and preoperative separate renal function grade (normal, moderate, and severe). The analyses revealed preoperative severe deterioration of separate renal function as the single independent significant predictor for the postoperative deterioration of renal function (OR: 9.09, 95% CI: 4.007–20.624, *P* < 0.001).

**Figure 2:**
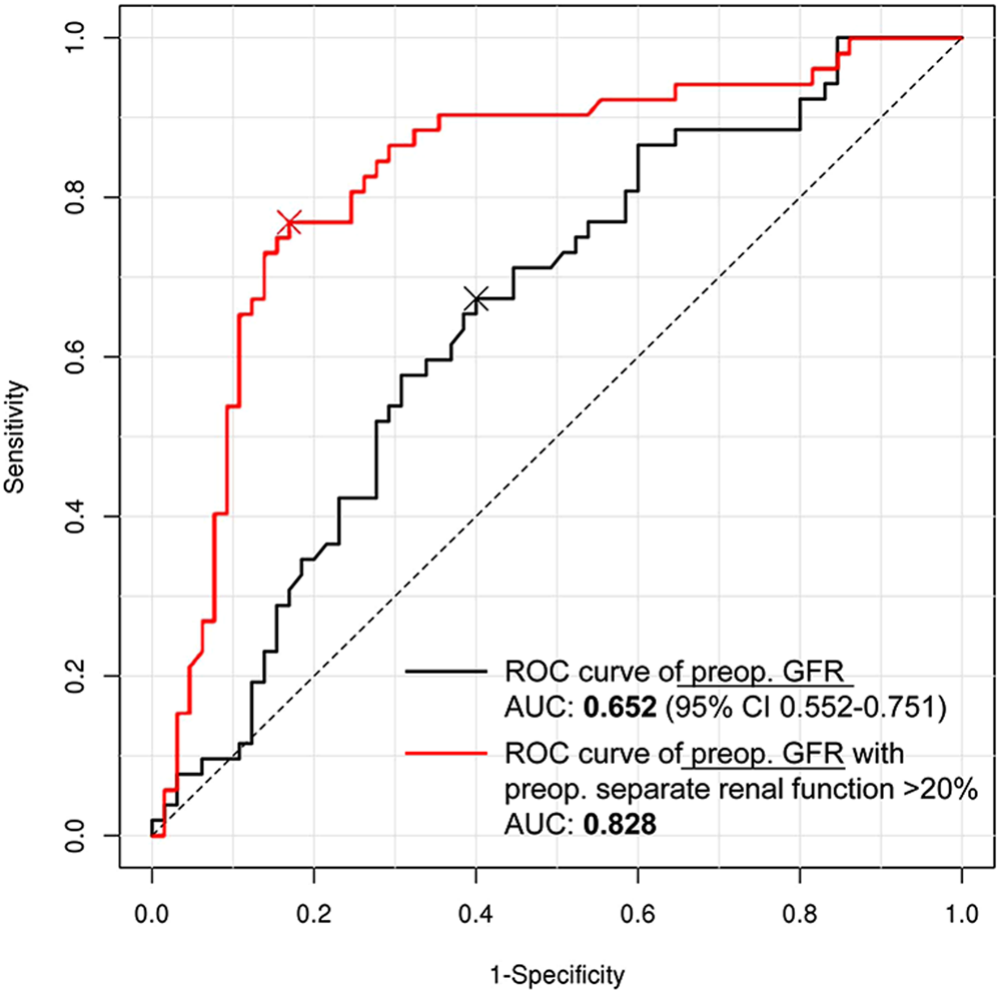
Receiver Operating Characteristic-curve analyses of the postoperative severe deterioration of separate function in the involved kidney. The black line represents univariate analysis for [preoperative GFR]. The red line represents bivariate analysis for [preoperative GFR] and preoperative deterioration of relative separate renal function more than 20% on the involved kidney. GFR = glomerular filtration rate; CI = confidence interval; ROC = receiver operating characteristic.

On the receiver operating characteristic (ROC) curve analysis, the area under the ROC curve (AUC) value for preoperative GFR was 0.652 (95% CI 0.552–0.751), and the optimal cutoff value was 78 mL/min/1.73 m^2^. When preoperative severe impairment of separation renal function was added, the AUC value was much improved to 0.828. In this bivariate analysis, the optimal cutoff value was 44 mL/min/1.73 m^2^ (Fig. 2).

## Discussion

In this study, severe deterioration of preoperative renal separate function to the extent greater than 20% led to an increase in the risk of worsening of postoperative renal separate function or condition of absence of recuperation. It has been reported that renal nephrolithiasis is one of the risk factors causing impairment in the function of the involved kidney^12,13^ and the damage can be reversed after treatment of calculi with improvement in renal function^14,15^. However, in the present study, it was observed that if the damage to the kidney was worse than a certain level, the deteriorated renal function was less likely to recover to normal condition. To the best of our knowledge, this is the first study to demonstrate perioperative changes in separate renal function according to the level of renal injury in a prospective database.

The probabilities of the presence of postoperative differences in separate renal function >20% were 76.2%, 24.1%, and 6.5% in preoperative severe, moderate, and normal separate renal function groups, respectively. On the other hand, the probabilities of the presence of postoperative normal separate renal function <10% were 4.8%, 37.9%, and 80.4% in preoperative severe, moderate, and normal separate renal function groups, respectively. Apparently, based on the result, it was conjectured that the kidneys will have less chance of postoperative improvement under a scenario of worsened preoperative renal function. The results of the present study correspond well with those of previous studies showing that moderate renal insufficiency of baseline serum creatinine >1.5 mg/dL or GFR < 10 mL/min/1.73 m^2^ of the corresponding kidney is a significant risk factor of deterioration of renal function after surgery^15,16^.

Previous studies have investigated changes in postoperative renal function using serum or urine markers^17^. However, serum creatinine level, estimated glomerular filtration rate, and urinary markers for tissue damage have the main drawback of being influenced by the compensatory effect of contralateral kidney, which might counterbalance a functional decline in the operated side^6^. In the present study, the mean creatinine level was 1.0 in the deterioration group. Though renal cortical thickness assessed through imaging study is known to be associated with poor renal outcome, quantification remains a difficult task. In addition, not every kidney with thin renal cortex is non-functional^18^. Therefore, measurement of separate renal function by DTPA renal scan is a more accurate method to confirm perioperative changes in renal function compared to laboratory results of serum or urine markers.

The results of this study indicated that perioperative separate renal functional data were necessary to evaluate accurate renal function. As the occurrence of bilateral obstructive uropathy caused by renal calculi is uncommon, it is hypothesized that a precise assessment of the functional status of each renoureteral unit, as well as estimation of the total renal function, will be helpful in selecting an optimal treatment and a management plan^19^. As per the AUA guidelines, a decrease in the renal function of the involved kidney may necessitate the employment of other therapeutic options ranging from observation to nephrectomy^20^. Additionally, establishing baseline renal function can be useful for following treatment outcomes of upper urinary tract stone disease^20^.

As per the AUA guidelines, for patients with asymptomatic non-obstructing caliceal stones, clinicians may offer active surveillance^20^. In EAU guideline, it is recommended that asymptomatic caliceal stones <15 mm can be observed for conservative treatment^1^. However, asymptomatic caliceal stones might have a higher probability of causing deterioration of renal function^7^. Based on our study results, an appropriate assessment of the separate function of the affected kidney is important before selecting active surveillance for renal calculi. In addition, in follow up evaluations, regular confirmation of absence of deterioration of separate renal function is needed for timely intervention for high-risk stone formers. The final goal of stone management is not only to achieve a stone-free status but also to preserve the renal function.

This study has some limitations which have to be pointed out. First, although the baseline hypertension was significantly higher in the moderate and severe deterioration groups, the incidence of hypertension did not change during the perioperative period and no meaningful information was found. The importance of the duration of obstruction on the revivability of preoperatively deteriorated renal function has been studied previously^21^. In this study, we were unable to confirm the duration of impacted calculi. Most of the patients were symptomatic and the duration of obstruction was not long with any decisive influence on the outcomes of this study.

In summary, preoperative severe deterioration of separate renal function was a significant predictor for the postoperative deterioration of renal function. Patients with the less preoperative deterioration of separate renal function had a high probability of regaining normal separate renal function. Consequently, early intervention is necessitated before severe aggravation of renal function.
