# Author Correction: Changes in separate renal function in patients who underwent minimally invasive renal stone surgery according to the preoperative functional deterioration

**DOI:** 10.1038/s41598-019-44432-8

**Published:** 2019-05-28

**Authors:** Min Soo Choo, Juhyun Park, Min Chul Cho, Hwancheol Son, Hyeon Jeong, Sung Yong Cho

**Affiliations:** 1 Department of Urology, Hallym University Dongtan Sacred Heart Hospital, Hwaseong, Korea; 2 Department of Urology, SMG-SNU Boramae Medical Center, Seoul, Korea; 3 Seoul National University Hospital, 101, Daehak-ro Jongno-gu, Seoul, 03080 South Korea

Correction to: *Scientific Reports* 10.1038/s41598-019-40485-x, published online 05 March 2019

The original version of this Article omitted an affiliation for Sung Yong Cho. The correct affiliations for Sung Yong Cho are listed below:

Department of Urology, SMG-SNU Boramae Medical Center, Seoul, Korea.

Seoul National University Hospital, 101, Daehak-ro Jongno-gu, Seoul, 03080, South Korea.

This error has now been corrected in the HTML and PDF versions of the Article.

